# Positional Therapy: A Real Opportunity in the Treatment of Obstructive Sleep Apnea? An Update from the Literature

**DOI:** 10.3390/life15081175

**Published:** 2025-07-24

**Authors:** Elvia Battaglia, Valentina Poletti, Eleonora Volpato, Paolo Banfi

**Affiliations:** 1 IRCCS Fondazione Don Carlo Gnocchi, 20148 Milan, Italy; ebattaglia@dongnocchi.it (E.B.); eleonora.volpato@unicatt.it (E.V.); pabanfi@dongnocchi.it (P.B.); 2 Department of Psychology, Università Cattolica del Sacro Cuore, 20123 Milan, Italy

**Keywords:** positional therapy, POSA, obstructive sleep apnea, sleep apnea, adherence to treatment

## Abstract

Obstructive sleep apnea (OSA) is a prevalent and heterogeneous sleep disorder associated with significant health and societal burdens. While continuous positive airway pressure (CPAP) remains the gold standard treatment, its limitations in adherence and patient tolerance have highlighted the need for alternative therapies. Positional therapy (PT), which targets apneas that occur predominantly in the supine position, has emerged as a promising option for individuals with positional OSA (POSA). This narrative review synthesizes the current literature on PT, examining its clinical indications, typologies, comparative efficacy with CPAP, oral appliances, and hypoglossal nerve stimulation, as well as data on adherence and barriers to long-term use. Traditional methods such as the tennis ball technique have largely been replaced by modern vibrotactile devices, which demonstrate improved comfort, adherence, and comparable short-term outcomes in selected POSA subjects. While PT remains inferior to CPAP in reducing overall AHI and oxygen desaturation, it performs favorably in terms of mean disease alleviation (MDA) and sleep continuity. Importantly, treatment effectiveness is influenced by both anatomical and non-anatomical traits, underscoring the need for accurate phenotyping and individualized care. PT should be considered within a broader patient-centered model that incorporates preferences, lifestyle, and motivational factors. Further research is needed to validate long-term efficacy, optimize selection criteria, and integrate PT into personalized OSA management strategies.

## 1. Introduction

Obstructive sleep apnea (OSA) syndrome is one of the most common sleep-disordered breathing (SDB) conditions. Over the last decades, numerous studies have highlighted the significant personal and societal costs associated with OSA, which is a major contributor to inadequate sleep, adversely affecting health, well-being, safety, and productivity [1]. Despite its clinical relevance, it is estimated that only about 40% of individuals affected by OSA(S) receive a formal diagnosis [2].

OSA is frequently associated with obesity, cardiometabolic comorbidities, and an elevated cardiovascular risk profile [3].

The gold standard treatment for OSA is continuous positive airway pressure (CPAP), yet its effectiveness is often undermined by low adherence and discomfort, thus highlighting the need for alternative treatment options [4].

In recent years, the understanding of OSA has evolved significantly, with growing evidence suggesting that it encompasses a spectrum of clinical and pathophysiological phenotypes. This conceptual shift has opened new perspectives for the development of tailored, phenotype-based interventions [5].

Among the most described clinical clusters are individuals with excessive daytime sleepiness (EDS), those with disturbed sleep, and minimally symptomatic individuals [6].

From a pathophysiological standpoint, in addition to upper airway anatomical compromise, a variety of non-anatomical traits have been identified—such as ventilatory control instability, arousal threshold, and upper airway muscle responsiveness—that support a more personalized, multimodal approach to treatment [5].

Currently, the decision-making process for OSA therapy goes beyond evidence of treatment efficacy alone and increasingly integrates considerations such as patient phenotype, treatment availability, cost, and acceptance.

Positional therapy (PT), which targets a specific subgroup of individuals whose apneic events are position-dependent, has emerged as one such promising alternative [1].

In this context, the present narrative review aims to provide an updated and critical synthesis of the literature on positional therapy (PT) to identify which patient profiles may benefit most from this treatment option.

Particular attention is given to defining relevant phenotypes, clinical characteristics, types of positional devices available, and issues related to treatment compliance.

By summarizing the evolving body of evidence and highlighting areas of consensus as well as ongoing debate, this review seeks to support clinicians in the personalized management of OSA and to inform future research directions in the field.

## 2. Materials and Methods

Given the increasing interest in PT for the management of OSA, and the substantial heterogeneity across existing studies in terms of study populations, definitions, and outcome measures, a narrative review was considered the most appropriate methodological approach. Unlike systematic reviews, which rely on strict inclusion criteria and standardized data synthesis, narrative reviews offer greater flexibility in addressing complex and evolving topics. This is particularly relevant when the available evidence spans diverse domains—clinical, physiological, and technological—and exhibits marked methodological variability. Such an approach is well-suited to explore inter-study differences in patient phenotypes, device characteristics, and adherence patterns, which are often insufficiently standardized across the current literature [7].

This review seeks to elucidate the clinical, pathophysiological, and behavioral factors that modulate the efficacy and applicability of PT in the management of obstructive sleep apnea OSA. Specifically, the objectives were to: (1) characterize the clinical and phenotypic profiles of patients most likely to benefit from PT; (2) provide an overview of currently available positional devices and their mechanisms of action; (3) compare PT with both standard and alternative treatment modalities for OSA; and (4) synthesize current evidence regarding both treatment adherence and long-term adherence.

### Search Strategy

A non-systematic literature search was conducted using the following electronic databases: PubMed, Scopus, and the Cochrane Library. The search strategy combined keywords such as “Positional Therapy”, “POSA”, “Sleep Apnea”, and “Adherence to Treatment”. Boolean operators were used to enhance the sensitivity of the search and identify relevant studies across different disciplines and study designs. Table 1 reports the search summary.

## 3. Results

### 3.1. From Generic OSA to Positional OSA: Definitions and Classification Systems

OSA is an SBD characterized by repeated episodes of hypopnea or apnea due to upper airway obstruction during sleep.

The term OSA refers to the objective findings from sleep studies, while obstructive sleep apnea syndrome (OSA(S)) includes both the physiological events and associated clinical symptoms such as daytime sleepiness [8].

According to the American Academy of Sleep Medicine (AASM) [9], diagnosis and severity classification of OSA are based on the apnea–hypopnea index (AHI), which represents the average number of apneas and hypopneas per hour of sleep.

An AHI < 5 is considered normal (or “simple snoring” if snoring is present), mild OSA is defined as ≥5 to <15 events/h, moderate as ≥15 to <30 events/h, and severe as ≥30 events/h OSA is positively associated with male sex, age, and risk factors such as obesity [10].

Multiple studies have linked OSA(S) to increased risk of type 2 diabetes [11], certain malignancies [12], and cardiovascular and cerebrovascular diseases [13].

Furthermore, OSA(S) is associated with reduced quality of life (QoL) [14], impaired occupational functioning, and heightened risk of traffic accidents [15].

Epidemiological data indicate that OSA affects approximately 3–7% of adult males and 2–5% of adult females in the general population [16,17]. However, estimates vary due to methodological inconsistencies and difficulties in characterizing the syndrome. Alarmingly, only about 40% of individuals with OSA receive a diagnosis, leading to underestimation of its true prevalence and burden. Given its high prevalence and underdiagnosis, the clinical and economic impact of OSA is substantial [18].

In OSA patients, the frequency and duration of respiratory events can be influenced by body position. Individuals who exhibit a marked variation in AHI between sleeping postures are classified as having POSA [19].

Various classification systems for POSA have been proposed. The earliest and most widely used is Cartwright’s definition [20], which identifies POSA as a ≥50% reduction in AHI between supine and non-supine positions. However, this definition does not account for the duration of time spent in each position. As a result, patients who spend only brief periods in a non-supine posture may be misclassified as POSA.

To address this limitation, modified criteria and alternative systems have been developed [21,22,23]. Bignold et al. [23] proposed that to qualify as POSA, patients must have ≥20 min of sleep in both supine and non-supine positions and demonstrate an AHI reduction > 50% in the non-supine posture.

More recently, the Amsterdam Positional OSA Classification (APOC) was introduced [24].This system incorporates total sleep time (TST) in both the worst sleeping position (WSP, typically supine) and the best sleeping position (BSP, typically lateral), along with the magnitude of AHI reduction in the BSP.

The APOC system has been shown to enhance the identification of POSA patients and assist in determining appropriate candidates for PT.

A precise classification of POSA is not only essential for differential diagnosis but also serves as the cornerstone for appropriate prescription of PT. Indeed, the clinical effectiveness of PT strongly depends on correctly identifying positional phenotypes, thereby guiding clinicians in selecting patients who are most likely to benefit from this non-invasive treatment approach.

Table 2 reports a comparison of POSA classification systems.

### 3.2. Clinical and Pathophysiological Features of Positional OSA

#### 3.2.1. Prevalence, Clinical Profile, and Subtypes of POSA

POSA represents a distinct phenotype of OSA in which respiratory events predominantly occur during supine sleep [25]. When these events are confined exclusively to the supine position, the condition is referred to as exclusive POSA (e-POSA), defined by a supine-to-non-supine AHI ratio > 2:1 and a non-supine AHI < 5 events/h [25].

Prevalence estimates vary depending on the classification criteria used, with reported rates of 20–75% for POSA and 5.4–36% for e-POSA among patients with mild-to-severe OSA [26]. Such variability is attributed to differences in sample size, ethnicity [26], and the use of various classification systems—namely Cartwright, Bignold, and the APOC.

For example, in a Korean cohort of 1170 OSA patients, Mo et al. [27] reported a POSA prevalence of nearly 75%. This high rate in East Asian populations may relate to anatomical features such as a short cranial base and retrognathia, contributing to increased upper airway collapsibility in the supine position [28].

In a Swiss population-based study (n = 1719), Heinzer et al. [29] found POSA and e-POSA prevalence rates of 53% and 26%, respectively.

Iannella et al. [1], using three classification criteria in a cohort of 434 older patients, reported variable POSA prevalence: 49.3% (Cartwright), 20.5% (Bignold), and 22.6%, 38.9%, and 5.4% for APOC subclasses 1, 2, and 3, respectively.

Clinically, POSA patients are more frequently younger, male, less obese, and present with fewer symptoms, lower neck circumference, and fewer comorbidities [30]. Desaturations, heart rate variability, loud snoring, and apneas in this group occur almost exclusively in the supine position [31].

In a large French cohort (n = 8243) evaluated via polysomnography (PSG) or Home Sleep Apnea Test (HSAT), lower AHI, lower body mass index (BMI), younger age, and male sex were independently associated with POSA/e-POSA [26].

POSA is typically associated with mild-to-moderate OSA, while e-POSA often presents with mild severity [26]. Older patients, in contrast, tend to have more severe and non-positional OSA (NPOSA), supporting the hypothesis that supine-predominant OSA may represent an early stage in the natural progression of the disorder. Longitudinal data [32] showed that over 6.6 years, 30% of POSA patients transitioned to NPOSA due to increased BMI and AHI, while 70% remained stable.

#### 3.2.2. Positional Influence, Sleep Architecture, and Pathophysiology

Diagnostic modality can influence sleep position and thereby affect POSA classification. Cartwright et al. [33] found that PSG may constrain patients due to sensor setup, resulting in increased supine sleep. Metersky et al. [34] corroborated this, reporting 56% more supine sleep during PSG compared to HSAT. However, Kukwa et al. [35], analyzing 445 PSG and 416 HSAT recordings, found no significant difference. Notably, women had more supine sleep during HSAT than PSG.

Compared to NPOSA, POSA and e-POSA patients spend more time in the supine position, with e-POSA showing the most marked difference. Time spent supine is a key determinant of overall AHI. Both POSA and e-POSA patients also display lower desaturation indices and higher hypopnea prevalence [36], possibly reflecting mild upper airway structural changes that preferentially induce hypopneas during supine sleep. While some studies have reported small yet significant reductions in pharyngeal cross-sectional areas during supine sleep [37], others have not confirmed this [38].

POSA patients exhibit better sleep efficiency, reduced wakefulness after sleep onset (WASO), and lower arousal indices, along with increased N3 and REM sleep compared to NPOSA [39]. These findings may partially reflect their younger age. However, age-adjusted analyses [39] confirmed significant differences in sleep efficiency (+4.53, *p* = 0.018), N3 (+6.3, *p* < 0.001), and REM sleep (+3.8, *p* < 0.001). POSA patients were also less hypertensive, less obese, and scored lower on Mallampati, Berlin, and STOP questionnaires. These findings align with previous studies showing age and BMI as predictive factors [40], potentially explained by pharyngeal anatomical changes and reduced positional shifts with age.

Although POSA is generally associated with a milder disease profile, this does not consistently translate into fewer daytime symptoms. In a cross-sectional study involving 1052 adults, Lee et al. [25] reported that individuals with POSA exhibited significantly lower AHI, Respiratory Disturbance Index (RDI), Oxygen Desaturation Index (ODI), higher minimum SaO_2_, lower BMI, older age, fewer cases of type 2 diabetes, and improved sleep architecture compared to NPOSA [41].

Nevertheless, no differences were found in Epworth Sleepiness Scale (ESS), Beck Depression Inventory (BDI), State-Trait Anxiety Inventory—State subscale (STAI-S), or Short Form-36 Health Survey (SF-36). Similar findings were confirmed in subtype analyses and by other authors [42,43], suggesting that sleepiness and QoL measures do not significantly differ between groups.

The pathophysiology of POSA is multifactorial and extends beyond anatomical narrowing. Non-anatomical traits—including reduced upper airway gain [44,45,46], elevated loop gain [46], low arousal threshold [47], and decreased lung volume [48]—contribute to variability in positional susceptibility.

Joosten et al. [49] examined these traits in patients with severe OSA and showed that lateral positioning significantly increased Functional Residual Capacity (FRC), passive and active ventilation (V0), and decreased Critical Closing Pressure (Pcrit). However, loop gain and arousal threshold were unaffected.

In individuals with supine-predominant OSA (supine-to-non-supine AHI > 4:1) [50], lateral sleep improved Upper Airway Gain (UAG) and Pcrit and showed a trend toward higher active V0. This subgroup appears to maintain airway patency more effectively in lateral positions due to superior upper airway dilator muscle function. These changes likely result from increased caudal tracheal traction, anatomical repositioning of the lateral pharyngeal walls, and altered gravitational vectors.

While the genioglossus muscle is more active in the supine position [51], lateral positioning may improve its mechanical efficiency by reducing passive collapse. Joosten et al. [52] confirmed that patients with supine-predominant OSA had significant improvements in UAG and near-significant improvements in active V0 (*p* = 0.052) in the lateral position, despite stable passive V0. Increased FRC in lateral posture, as observed by other authors [53], may reduce airway collapsibility via enhanced tracheal traction and lower surrounding tissue pressure [54]. Furthermore, the elliptical shape of the velopharynx in supine sleep is prone to collapse at the lateral walls [55]; this shape becomes more circular in the lateral position, increasing stability.

Body position significantly influences upper airway patency because of gravity on pharyngeal structures. In the supine position, the tongue and soft palate tend to collapse posteriorly into the airway, contributing to obstruction [37]. In contrast, during lateral sleep, these structures are oriented perpendicularly to the gravitational vector, resulting in reduced airway collapsibility.

These positional dynamics help explain why certain individuals—including those with moderate-to-severe OSA—may derive substantial benefit from positional therapy (PT). While PT alone may be insufficient for all individuals with severe OSA, its combination with interventions targeting non-anatomical pathophysiological traits—such as elevated loop gain or a low arousal threshold—may represent a viable alternative to CPAP in selected cases [49].

### 3.3. Positional Therapy for POSA: Types of Treatment

#### 3.3.1. Traditional Approaches: From Tennis Ball Technique to Positional Aids

PT refers to any intervention designed to avoid the worst sleeping position—typically the supine position—in patients with POSA. The most widely known and historically used method is the Tennis Ball Technique (TBT) [56]. It involves sewing a pocket into the back of the patient’s sleepwear to hold a tennis-ball-sized object, which causes discomfort when lying on the back, thereby encouraging lateral sleeping.

Over time, various adaptations of the TBT have been introduced. In a single-arm study, De Vries and colleagues [55] evaluated its efficacy in 53 POSA patients, 40 of whom underwent follow-up polysomnography. Short-term results showed that PT with the TBT—whether using a commercial waistband or a self-made device—effectively reduced the time spent in the supine position. AHI significantly improved (from 14.5 to 5.9 events/h, *p* < 0.001), as did EDS. PT was effective in 68% of patients. BMI remained stable throughout the study, suggesting that the observed benefits were attributable to PT rather than weight loss. Notably, in four patients, AHI worsened despite a reduction in supine sleep time, likely due to an increased number of respiratory events in the non-supine position.

Alternative postural interventions have also been investigated. Zuberi et al. [57] assessed the efficacy of the SONA Pillow^®^, a triangular inclined pillow designed to facilitate lateral sleep by allowing the arm to be placed beneath the head. In a cohort of 22 individuals with mild to moderate OSA, use of the pillow led to a significant decrease in the RDI, from an average of 17 to fewer than five events per hour (*p* < 0.0001), especially during REM sleep (*p* = 0.001). SaO_2_ levels significantly improved (*p* = 0.004), and snoring was reduced or eliminated in most participants (*p* = 0.017).

Similarly, Bidarian-Moniri et al. [58] demonstrated that prone positioning using a combination of a specially designed mattress and pillow resulted in an overall reduction in AHI among OSA patients.

#### 3.3.2. Technological Advances: Vibrotactile Positional Devices

In recent years, technological innovation has spurred the development of more sophisticated vibrotactile PT devices, designed to overcome the limitations of traditional methods. These include the Night Shift^®^ [59], the Sleep Position Trainer (SPT^®^) [60], the BuzzPOD^®^ [61], and the Somnibel, Sibelmed^®^ [62]. The Night Shift^®^ is a small wearable device secured around the neck or chest, using a magnetic clasp or strap. The SPT^®^ and BuzzPOD^®^ are worn on the chest and fastened with a strap. All three devices use built-in accelerometers to detect body or head position. Upon detecting a supine posture, they deliver progressively stronger vibrations until the user changes position. Of these, only the Night Shift^®^ has received FDA approval for the treatment of POSA.

Initial clinical studies have demonstrated promising outcomes. In a randomized crossover trial, Bignold et al. [23] showed that one week of treatment with a vibrotactile device significantly reduced both the proportion of supine sleep and mean AHI (from 25 to 13.7 events/h, *p* = 0.03). Van Maanen et al. [63], in a single-arm study of 31 patients, reported significant reductions in supine sleep and median AHI, as well as improvements in ESS and Functional Outcomes of Sleep Questionnaire (FOSQ) scores.

A randomized controlled trial conducted by Laub et al. [64] confirmed that these improvements in AHI and daytime sleepiness persisted after six months of SPT^®^ use. Similarly, van Maanen and Levendowski demonstrated significant AHI reductions and improved sleep architecture and mood symptoms (PHQ-9 score, *p* = 0.027) following four weeks of Night Shift^®^ therapy.

In a head-to-head comparison of SPT and TBT, Eijsvogel et al. [65] found that both interventions reduced supine sleep to a median of 0%, though the range was wider in the SPT group. Treatment success (defined as AHI < 5 events/h) was higher in the SPT group (68.0%) compared to TBT (42.9%), though the difference did not reach statistical significance. SPT was superior in improving sleep quality, total QSQ scores, and the QSQ subdomains related to nocturnal symptoms and social interaction.

A systematic review [66] evaluated the efficacy of vibrotactile devices, including the Night Shift^®^, SPT^®^, BuzzPOD^®^, and Somnibel^®^. The review confirmed that these devices significantly reduced both AHI and time spent in the supine position. While small improvements were noted in ESS and FOSQ scores, these did not exceed the minimal clinically important difference (MCID). Sleep efficiency and arousal index showed minimal changes, and generic quality of life measures (e.g., SF-36) did not significantly improve. More recently, Gao and collaborators conducted a comparative meta-analysis and demonstrated that although CPAP remains the most effective treatment in reducing overall AHI, sleep positional therapy (SPT) represents a safer and better-tolerated alternative. In particular, SPT was associated with significant reductions in supine AHI and a lower incidence of device-related adverse events, especially among patients who are intolerant to CPAP [67].

A recent randomized controlled trial further supported the efficacy of Somnibel in a three-arm study design. The forehead-worn vibrating device led to a significant reduction in AHI and time spent in the supine position, along with improvements in sleep architecture, specifically in N3 and REM stages, without increasing arousals. These results suggest that Somnibel may be particularly effective in enhancing sleep quality in POSA patients without compromising comfort or adherence [62].

Concerns about vibration-induced arousals were not supported by the data, as sleep efficiency and arousal index remained largely unaffected. Though the ESS improved, the change did not meet the threshold for clinical relevance [68]. Likewise, improvements in FOSQ scores did not surpass the MCID of 1.8 points [69].

In summary, vibrotactile positional devices represent a significant advancement in the management of POSA. They are generally well tolerated and effective in reducing supine-related respiratory events. However, their long-term impact on quality of life and daytime functioning remains an area for further research.

Table 3 reports a summary of types of POSA treatment.

### 3.4. Efficacy of Positional Therapy Compared to Other Treatments

#### 3.4.1. Comparison with CPAP

The effectiveness of PT versus CPAP has been investigated in several studies. In a crossover trial [70], 38 patients with POSA underwent sleep studies with both CPAP and a foam device worn on the back. Results showed similar efficacy between treatments in reducing AHI to <5 events/h (92% vs. 97%, *p* = 0.16).

Although no RCTs have yet compared vibratory PT devices directly to CPAP, ongoing studies are addressing this gap. The POSAtive study [71], a European multicenter RCT, is comparing the NightBalance Sleep Position Trainer (SPT) and auto-adjusting CPAP (auto-CPAP) in 120 POSA patients, focusing on AHI reduction and adherence over two 6-week periods.

In Australia, the SUPA OSA Trial [72] compares the BuzzPOD device with CPAP in 140 patients (originally 280), also using a crossover design. The main endpoint is a reduction in daytime sleepiness (ESS ≥ 8).

#### 3.4.2. Comparison with Oral Appliance Therapy (OAT)

Benoist et al. [73] compared SPT and mandibular advancement devices (OAT) in 99 POSA patients. Both significantly reduced AHI (SPT: 13.0 to 7.0/h; OAT: 11.7 to 9.1/h), with no difference between groups. Adherence was higher for SPT in intention-to-treat analysis (88.4% vs. 60.5%), but mean disease alleviation was similar.

A meta-analysis [74] found OAT reduced non-supine AHI and ESS scores more than PT (*p* = 0.0006 and *p* = 0.0009, respectively), while PT reduced supine sleep more (*p* = 0.0001). No differences emerged for total or supine AHI, ODI, sleep efficiency, arousal index, FOSQ, adherence, or SpO_2_.

Contrastingly, Suzuki et al. [75] reported greater reductions in supine sleep and respiratory events with PT, and better deep sleep improvement.

Marciuc et al. [76] confirmed OAT’s efficacy in improving AHI. Marklund et al. [77] found higher treatment success in women (OR = 2.4) and POSA men (OR = 6.0 vs. non-POSA). Makihara et al. [78] showed that both 50% and 75% mandibular advancement improved AHI and AI, but ESS remained unchanged.

Gao et al. [67] found no significant difference between SPT and OAT for reducing total or supine AHI, though SPT significantly lowered arousal index (−7.11; 95% CI: −10.52 to −3.71), indicating better sleep continuity. N3 sleep improved with SPT, but not REM or N1. Further research is needed to assess clinical relevance.

No significant differences in oxygen saturation (mean or minimum SaO_2_) were found among SPT, placebo, OAT, or CPAP [79]. SPT may not mitigate hypoxic burden. ODI was significantly higher in the SPT group vs. placebo, suggesting that SPT might not suit patients with severe desaturations.

#### 3.4.3. Hypoglossal Nerve Stimulation (HGNS)

Evidence on HGNS in POSA remains limited. In a retrospective study [80], authors found significant AHI and ESS reductions in both POSA and non-POSA individuals following HGNS (AHI: 21.5 to 6.15, *p* < 0.001; ESS: 7.5 to 4.5, *p* < 0.03).

However, White et al. [81] reported that 76% of people with OSA pre-implantation continued to exhibit POSA post-HGNS, despite AHI improvements. These results suggest HGNS may not resolve positional dependency, highlighting the need for adjunctive PT in symptomatic individuals.

#### 3.4.4. Combination Therapy: MAD and PT

Dieltjens et al. [82] examined the combined use of mandibular advancement device (MAD) and SPT in those with residual supine-predominant OSA. A two-night PSG showed that the combined approach reduced median AHI from 20.9 to 5.5/h, outperforming MAD alone (11.6/h) and SPT alone (12.8/h). With combination therapy, 95% of subjects achieved ≥50% AHI reduction.

### 3.5. Adherence to Positional Therapy: Prevalence and Barriers

Adherence constitutes a crucial determinant of the clinical effectiveness of PT. Although short-term compliance rates are generally acceptable, evidence regarding long-term adherence remains limited and heterogeneous. One study reported that only 41.6% of patients maintained regular use at six months, with higher adherence and more favorable therapeutic outcomes observed in individuals with mild-to-moderate OSA compared to those with severe disease [5].

A systematic review and meta-analysis conducted by Ravesloot et al. [83] offers a comprehensive overview of adherence patterns with new-generation vibrotactile PT devices. The authors found high short-term (1-month) compliance rates, ranging from 76% to 96%, when compliance was defined as ≥4 h per night, seven nights per week [60,61,65]. These figures suggest promising early acceptance and use of modern PT technologies.

Van Maanen et al. [84] also reported on longer-term adherence, although interpretation of these findings is complicated by a substantial 50% loss to follow-up. During the initial three-week period, average nightly use was 6.8 h, while the median use over one month and six months was 6.5 and 5.5 h per night, respectively. At one month, between 92.7% and 96% of individuals met the defined compliance threshold. In support of these findings, a prospective study [85] demonstrated the long-term efficacy and adherence of a neck-based vibrotactile device, reporting sustained clinical benefits and favorable tolerance up to 12 months of follow-up.

To enable direct comparison between OSA treatment modalities, Eijsvogel et al. [65] proposed the use of Mean Disease Alleviation (MDA), a composite metric reflecting both therapeutic efficacy (reduction in AHI) and adjusted compliance (based on actual device use relative to total sleep time). For mandibular repositioning devices (MRD) and CPAP, previously published MDA values approximate 50% [86]. In their study, the SPT achieved a significantly higher MDA of 70.5%, compared to 48.6% for the traditional TBT [87]. This superior outcome for SPT was attributed to both high therapeutic efficacy and excellent adjusted compliance.

Interestingly, although the SPT was effective in preventing supine sleep, not all vibratory stimuli were followed by a positional change. The device manufacturer later clarified that the vibratory signal in older models was programmed to cease after 30 s; this limitation has been addressed in subsequent generations of the device. Table 4 reports adherence rates and definitions described by included studies, while Table 5 highlights drawbacks and limitations of PT.

## 4. Discussion

This narrative review highlights PT as a promising and underutilized option in the tailored management of OSA, particularly in people with POSA. Our findings support the idea that, when appropriately prescribed based on phenotype-specific criteria, PT can serve as an effective and well-tolerated alternative or adjunct to conventional therapies, such as CPAP and OAT.

Compared to traditional techniques such as the TBT, modern vibrotactile devices demonstrate improved adherence, comfort, and efficacy. Although CPAP remains the gold standard due to its robust impact on AHI reduction, growing evidence suggests that in selected individuals with POSA, PT may achieve comparable short-term outcomes in AHI normalization. Furthermore, when compared to OAT, PT appears to offer advantages in sleep continuity (e.g., lower arousal index), though its efficacy on oxygenation remains variable and warrants caution in individuals with significant desaturation profiles.

Importantly, the concept of MDA has emerged as a clinically meaningful metric that integrates both efficacy and adherence. The higher MDA observed with vibrotactile PT devices relative to TBT and comparable to CPAP underscores the need to consider not only how effective a therapy is under ideal conditions, but how effectively it is used in real-world settings.

Nevertheless, data on long-term adherence remain limited, and drop-out rates pose a critical barrier to widespread implementation.

Moreover, PT is not universally effective: both anatomical and non-anatomical traits influence outcomes, and roughly half of individuals in some cohorts do not respond adequately. This highlights the importance of refined people selection, potentially integrating physiological endotyping.

From a broader clinical and psychosocial perspective, PT aligns with contemporary calls for a more patient-centered approach in sleep medicine. Adherence should not be viewed merely as a behavioral variable, but should be recognized as deeply influenced by patient preferences, comfort, lifestyle, and motivation. Engaging with patients’ experiences, identifying barriers to therapy acceptance, and incorporating their perspectives into shared decision-making are essential components [88]. The psychological burden of chronic therapy, especially with devices perceived as restrictive or intrusive, must be weighed against therapeutic benefits. In this light, shared decision-making and motivational support play a pivotal role in enhancing both adherence and patient satisfaction [89].

## 5. Conclusions

Choosing the most appropriate treatment for each individual with OSA remains a clinical challenge. Distinct phenotypic and endotypic profiles exist, and therapeutic decisions should be guided not only by disease severity and positional dependency, but also by patient preferences and acceptance.

Future research should prioritize long-term effectiveness, combination strategies, and broader patient-centered outcomes, including mood, cognition, and quality of life. Large-scale, real-world studies are needed to refine adherence profiles, evaluate cost-effectiveness, and identify psychological and behavioral predictors of sustained use. Clinicians are encouraged to implement regular follow-up and objective outcome monitoring for all treatment types—CPAP or non-CPAP—ensuring timely adjustments when necessary.

Ultimately, positional therapy represents an important addition to the therapeutic toolbox for OSA. When accurately matched to the appropriate phenotype and integrated into a holistic, personalized care model, it may improve adherence, enhance patient satisfaction, and expand individualized treatment pathways.

## Figures and Tables

**Table 1:** Summary of the literature search strategy.

Items	Specification
Date of search (specified to date, month, and year)	Up to June 2025 (exact search date not specified).
Databases and other sources searched	PubMed, Scopus, and the Cochrane Library.
Search terms used (including MeSH and free-text search terms and filters)	A combination of free-text keywords and Boolean operators was used: “Positional Therapy”, “POSA”, “Sleep Apnea”, and “Adherence to Treatment”.
Timeframe	No explicit time restrictions were applied; studies published up to June 2025 were included.
Inclusion and exclusion criteria (study type, language restrictions etc.)	Inclusion criteria: (1) studies conducted on adult patients with OSA or positional OSA (POSA); (2) studies investigating the use, effectiveness, or adherence to positional therapy; (3) articles published in English, peer-reviewed, and open-access. Exclusion criteria: non-English, non-peer-reviewed, or non-open-access studies.
Selection process (who conducted the selection, whether it was conducted independently, how consensus was obtained, etc.)	The selection process was not conducted using a structured or independent method. As this was a narrative review, study selection was performed by the authors based on relevance to the review objectives. No formal consensus procedure or dual screening is reported.
Any additional considerations, if applicable	A narrative review methodology was chosen due to the heterogeneity of existing studies in terms of patient populations, definitions, device types, and outcome measures. The flexible structure allowed for the exploration of complex, evolving, and interdisciplinary topics relevant to clinical and behavioral aspects of positional therapy.

**Table 2:** POSA classification systems.

Classification System	Definition	Strengths	Limitations
Cartwright	≥50% reduction in AHI in supine vs. non-supine	Simple; widely used	Does not consider time spent in each position
Bignold	≥50% reduction in AHI and ≥20 min in both positions	More accurate; considers time distribution	Still binary; less commonly used
APOC	Uses BSP/WSP, AHI reduction, and sleep time distribution	Multidimensional; guides therapy decisions	More complex; newer and less validated

**Table 3:** General characteristics of main positional therapy devices for POSA.

Device	Type	Placement	Mechanism of Action
Tennis Ball Technique (TBT)	Traditional (manual)	Sewn into the back of nightwear	Causes discomfort in supine position, prompting lateral repositioning
SONA Pillow^®^	Positional (inclined pillow)	Under head and arm on the bed	Encourages side-sleeping through an inclined surface and arm support
Prone Pillow System	Positional (mattress + pillow)	Full-body bed setup	Facilitates prone positioning through ergonomic mattress and pillow configuration
Night Shift^®^	Vibrotactile (electronic)	Neck or chest	Delivers escalating vibrations when supine posture is detected via accelerometer
Sleep Position Trainer (SPT^®^)	Vibrotactile (electronic)	Chest	Provides vibration stimulus to discourage supine position through body position sensor
BuzzPOD^®^	Vibrotactile (electronic)	Chest	Detects supine posture and delivers vibratory feedback to induce lateral repositioning
Somnibel^®^	Vibrotactile (electronic)	Forehead	Uses a head-position accelerometer to detect supine posture and emits progressive vibrations to encourage repositioning without disturbing sleep

**Table 4:** Summary of long-term adherence to vibrotactile positional therapy devices.

Study	Device Type/Placement	Follow-Up Duration	Adherence Definition	Reported Adherence and Outcomes
Gambino et al. (2022) [5]	General PT (unspecified)	6 months	Regular use (not numerically defined)	41.6% maintained regular use at 6 months; higher adherence and better outcomes in mild–moderate OSA
Ravesloot et al. (2017) [22]	Vibrotactile (various devices)	1 month	≥4 h/night, 7 nights/week	Adherence ranged from 76% to 96% across devices
van Maanen et al. (2013) [60]	Sleep Position Trainer (SPT^®^), chest	3 weeks, 1 mo, 6 mo	Median nightly use and compliance threshold	6.8 h (3 wks), 6.5 h (1 mo), 5.5 h (6 mo); 92.7–96% met ≥ 4 h/night threshold
De Corso et al. (2020) [85]	Neck-based vibrotactile device	12 months	Regular use (not numerically defined)	Sustained clinical efficacy and favorable tolerance up to 12 months

**Table 5:** Reported drawbacks and limitations of positional therapy.

Type of PT	Drawback/Side Effect	Details/Clinical Implications
Tennis Ball Technique (TBT)	Discomfort and poor tolerability	Causes discomfort in supine position, potentially leading to poor sleep quality and low adherence
	Limited effectiveness in some patients	In 32% of patients, therapy was ineffective; in some, AHI worsened despite reduced supine time
Vibrotactile devices	Non-response to stimuli	Not all vibratory stimuli triggered postural change; desensitization or habituation possible
	Early models had suboptimal programming	Vibration stopped after 30 s in older SPT models, reducing efficacy
	Mild or clinically irrelevant symptom improvements	Improvements in ESS and FOSQ did not exceed MCID thresholds
General limitations	Limited long-term adherence	Regular use at 6 months dropped to 41.6% in some studies; dropouts common
	Potential unsuitability in severe OSA	PT may not sufficiently reduce oxygen desaturations; ODI remained higher than in control groups
	Lack of impact on generic quality of life	Minimal or no improvement observed in general QoL scales (e.g., SF-36)

## References

[1] Iannella G., Magliulo G., Lo Iacono C.A.M., Bianchi G., Polimeni A., Greco A., De Vito A., Meccariello G., Cammaroto G., Gobbi R. (2020). Positional Obstructive Sleep Apnea Syndrome in Elderly Patients. Int. J. Environ. Res. Public Health.

[2] Kapur V.K., Auckley D.H., Chowdhuri S., Kuhlmann D.C., Mehra R., Ramar K., Harrod C.G. (2017). Clinical Practice Guideline for Diagnostic Testing for Adult Obstructive Sleep Apnea: An American Academy of Sleep Medicine Clinical Practice Guideline. J. Clin. Sleep Med..

[3] Bonsignore M.R., Baiamonte P., Mazzuca E., Castrogiovanni A., Marrone O. (2019). Obstructive sleep apnea and comorbidities: A dangerous liaison. Multidiscip. Respir. Med..

[4] Bakker J.P., Weaver T.E., Parthasarathy S., Aloia M.S. (2019). Adherence to CPAP: What Should We Be Aiming For, and How Can We Get There?. Chest.

[5] Gambino F., Zammuto M.M., Virzì A., Conti G., Bonsignore M.R. (2022). Treatment options in obstructive sleep apnea. Intern. Emerg. Med..

[6] Ye L., Pien G.W., Ratcliffe S.J., Björnsdottir E., Arnardottir E.S., Pack A.I., Benediktsdottir B., Gislason T. (2014). The different clinical faces of obstructive sleep apnoea: A cluster analysis. Eur. Respir. J..

[7] Rother E.T. (2007). Systematic literature review X narrative review. Acta Paul. Enferm..

[8] Patil S.P., Schneider H., Schwartz A.R., Smith P.L. (2007). Adult Obstructive Sleep Apnea: Pathophysiology and Diagnosis. Chest.

[9] Berry R.B., Brooks R., Gamaldo C., Harding S.M., Lloyd R.M., Quan S.F., Troester M.T., Vaughn B.V. (2017). AASM scoring manual updates for 2017 (version 2.4). J. Clin. Sleep Med..

[10] Young T., Peppard P.E., Gottlieb D.J. (2002). Epidemiology of obstructive sleep apnea: A population health perspective. Am. J. Respir. Crit. Care Med..

[11] Siddiquee A.T., Kim S., Thomas R.J., Lee M.H., Lee S.K., Shin C. (2023). Obstructive sleep apnoea and long-term risk of incident diabetes in the middle-aged and older general population. ERJ Open Res..

[12] Gozal D., Ham S.A., Mokhlesi B. (2016). Sleep Apnea and Cancer: Analysis of a Nationwide Population Sample. Sleep.

[13] Parish J., Chest P.L. (2003). Quality of life in bed partners of patients with obstructive sleep apnea or hypopnea after treatment with continuous positive airway pressure. Chest.

[14] Poletti V., Battaglia E.G., Banfi P., Volpato E. (2025). Effectiveness of continuous positive airway pressure therapy on romantic relationships and intimacy among individuals with obstructive sleep apnea: A systematic review and a meta-analysis. J. Sleep Res..

[15] Luzzi V., Mazur M., Guaragna M., Di Carlo G., Cotticelli L., Magliulo G., Marasca B., Pirro V., Di Giorgio G., Ndokaj A. (2022). Correlations of Obstructive Sleep Apnea Syndrome and Daytime Sleepiness with the Risk of Car Accidents in Adult Working Population: A Systematic Review and Meta-Analysis with a Gender-Based Approach. J. Clin. Med..

[16] Shochat T., Pillar G. (2003). Sleep apnoea in the older adult: Pathophysiology, epidemiology, consequences and management. Drugs Aging.

[17] Young T., Palta M., Dempsey J., Peppard P.E., Nieto F.J., Hla K.M. (2009). Burden of Sleep Apnea: Rationale, Design, and Major Findings of the Wisconsin Sleep Cohort Study. WMJ.

[18] Borsoi L., Armeni P., Donin G., Costa F., Ferini-Strambi L. (2022). The invisible costs of obstructive sleep apnea (OSA): Systematic review and cost-of-illness analysis. PLoS ONE.

[19] Ravesloot M.J.L., Van Maanen J.P., Dun L., De Vries N. (2013). The undervalued potential of positional therapy in position-dependent snoring and obstructive sleep apnea—A review of the literature. Sleep Breath..

[20] Cartwright R.D. (1984). Effect of Sleep Position on Sleep Apnea Severity. Sleep.

[21] Frank M.H., Ravesloot M.J.L., van Maanen J.P., Verhagen E., de Lange J., de Vries N. (2015). Positional OSA part 1: Towards a clinical classification system for position-dependent obstructive sleep apnoea. Sleep Breath..

[22] Ravesloot M.J.L., Frank M.H., van Maanen J.P., Verhagen E.A., de Lange J., de Vries N. (2016). Positional OSA part 2: Retrospective cohort analysis with a new classification system (APOC). Sleep Breath..

[23] Bignold J.J., Mercer J.D., Antic N.A., McEvoy R.D., Catcheside P.G. (2011). Accurate position monitoring and improved supine-dependent obstructive sleep apnea with a new position recording and supine avoidance device. J. Clin. Sleep Med..

[24] Duce B., Kulkas A., Langton C., Töyräs J., Hukins C. (2017). Amsterdam positional OSA classification: The AASM 2012 recommended hypopnoea criteria increases the number of positional therapy candidates. Sleep Breath..

[25] Lee S.A., Paek J.H., Chung Y.S., Kim W.S. (2017). Clinical features in patients with positional obstructive sleep apnea according to its subtypes. Sleep Breath..

[26] Sabil A.K., Blanchard M., Trzepizur W., Goupil F., Meslier N., Paris A., Pigeanne T., Priou P., Le Vaillant M., Gagnadoux F. (2020). Positional obstructive sleep apnea within a large multicenter French cohort: Prevalence, characteristics, and treatment outcomes. J. Clin. Sleep Med..

[27] Mo J.H., Lee C.H., Rhee C.S., Yoon I.Y., Kim J.W. (2011). Positional Dependency in Asian Patients With Obstructive Sleep Apnea and Its Implication for Hypertension. Arch. Otolaryngol. Neck Surg..

[28] Chou Y.T., Yang T.M., Lin C.K., Huang S.Y., Tsai Y.H., Chang J.F., Hou Y.J., Lin Y.C. (2017). Pay attention to treating a subgroup of positional obstructive sleep apnea patients. J. Formos. Med. Assoc..

[29] Heinzer R., Petitpierre N.J., Marti-Soler H., Haba-Rubio J. (2018). Prevalence and characteristics of positional sleep apnea in the HypnoLaus population-based cohort. Sleep Med..

[30] Laub R.R., Mikkelsen K.L., Tønnesen P. (2015). Prevalence of positional obstructive sleep apnea and patients characteristics using various definitions. Eur. Respir. J..

[31] Oksenberg A., Gadoth N. (2014). Are we missing a simple treatment for most adult sleep apnea patients? The avoidance of the supine sleep position. J. Sleep Res..

[32] Oksenberg A., Goizman V., Eitan E., Nasser K., Gadoth N., Leppänen T. (2020). Obstructive sleep apnea: Do positional patients become nonpositional patients with time?. Laryngoscope.

[33] Cartwright R.D., Lloyd S., Lilie J., Kravitz H. (1985). Sleep Position Training as Treatment for Sleep Apnea Syndrome: A Preliminary Study. Sleep.

[34] Metersky M.L., Castriotta R.J. (1996). The Effect of Polysomnography on Sleep Position: Possible Implications on the Diagnosis of Positional Obstructive Sleep Apnea. Respiration.

[35] Kukwa W., Łaba J., Lis T., Sobczyk K., Mitchell R.B., Młyńczak M. (2022). Supine sleep patterns as a part of phenotyping patients with sleep apnea—A pilot study. Sleep Breath..

[36] Leppänen T., Töyräs J., Muraja-Murro A., Kupari S., Tiihonen P., Mervaala E., Kulkas A. (2016). Length of Individual Apnea Events Is Increased by Supine Position and Modulated by Severity of Obstructive Sleep Apnea. Sleep Disord..

[37] Isono S., Tanaka A., Nishino T. (2002). Lateral position decreases collapsibility of the passive pharynx in patients with obstructive sleep apnea. Anesthesiology.

[38] Walsh J.H., Leigh M.S., Paduch A., Maddison K.J., Armstrong J.J., Sampson D.D., Hillman D.R., Eastwood P.R. (2008). Effect of body posture on pharyngeal shape and size in adults with and without obstructive sleep apnea. Sleep.

[39] Oulhaj A., Al Dhaheri S., Su B.B., Al-Houqani M. (2017). Discriminating between positional and non-positional obstructive sleep apnea using some clinical characteristics. Sleep Breath..

[40] Peppard P.E., Young T., Palta M., Skatrud J. (2000). Prospective Study of the Association between Sleep-Disordered Breathing and Hypertension. N. Engl. J. Med..

[41] Joosten S.A., Hamza K., Sands S., Turton A., Berger P., Hamilton G. (2012). Phenotypes of patients with mild to moderate obstructive sleep apnoea as confirmed by cluster analysis. Respirology.

[42] Mador M.J., Kufel T.J., Magalang U.J., Rajesh S.K., Watwe V., Grant B.J.B. (2005). Prevalence of positional sleep apnea in patients undergoing polysomnography. Chest.

[43] Teerapraipruk B., Chirakalwasan N., Simon R., Hirunwiwatkul P., Jaimchariyatam N., Desudchit T., Charakorn N., Wanlapakorn C. (2012). Clinical and polysomnographic data of positional sleep apnea and its predictors. Sleep Breath..

[44] Eckert D.J., Lo Y.L., Saboisky J.P., Jordan A.S., White D.P., Malhotra A. (2011). Sensorimotor function of the upper-airway muscles and respiratory sensory processing in untreated obstructive sleep apnea. J. Appl. Physiol..

[45] Jordan A.S., Wellman A., Heinzer R.C., Lo Y.L., Schory K., Dover L., Gautam S., Malhotra A., White D.P. (2007). Mechanisms used to restore ventilation after partial upper airway collapse during sleep in humans. Thorax.

[46] Wellman A., Malhotra A., Jordan A.S., Stevenson K.E., Gautam S., White D.P. (2008). Effect of oxygen in obstructive sleep apnea: Role of loop gain. Respir. Physiol. Neurobiol..

[47] Jordan A.S., Eckert D.J., Wellman A., Trinder J.A., Malhotra A., White D.P. (2011). Termination of respiratory events with and without cortical arousal in obstructive sleep apnea. Am. J. Respir. Crit. Care Med..

[48] Stanchina M.L., Malhotra A., Fogel R.B., Trinder J., Edwards J.K., Schory K., White D.P. (2003). The influence of lung volume on pharyngeal mechanics, collapsibility, and genioglossus muscle activation during sleep. Sleep.

[49] Joosten S.A., Edwards B.A., Wellman A., Turton A., Skuza E.M., Berger P.J., Hamilton G.S. (2015). The effect of body position on physiological factors that contribute to obstructive sleep apnea. Sleep.

[50] Otsuka R., Ono T., Ishiwata Y., Kuroda T. (2000). Respiratory-Related Genioglossus Electromyographic Activity in Response to Head Rotation and Changes in Body Position. Angle Orthod..

[51] Malhotra A., Trinder J., Fogel R., Stanchina M., Patel S.R., Schory K., Kleverlaan D., White D.P. (2004). Postural effects on pharyngeal protective reflex mechanisms. Sleep.

[52] Tagaito Y., Isono S., Remmers J.E., Tanaka A., Nishino T. (2007). Lung volume and collapsibility of the passive pharynx in patients with sleep-disordered breathing. J. Appl. Physiol..

[53] Kairaitis K., Byth K., Parikh R., Stavrinou R., Wheatley J.R., Amis T.C. (2007). Tracheal traction effects on upper airway patency in rabbits: The role of tissue pressure. Sleep.

[54] Schwab R.J., Pack A.I., Gupta K.B., Metzger L.J., Oh E., Getsy J.E., Hoffman E.A., Gefter W.B. (1996). Upper airway and soft tissue structural changes induced by CPAP in normal subjects. Am. J. Respir. Crit. Care Med..

[55] De Vries G.E., Hoekema A., Doff M.H.J., Kerstjens H.A.M., Meijer P.M., Van Der Hoeven J.H., Wijkstra P.J. (2015). Usage of positional therapy in adults with obstructive sleep apnea. J. Clin. Sleep Med..

[56] Wali S.O., AlQassas I., Qanash S., Mufti H., Alamoudi M., Alnowaiser M., Alnowaiser M., Bakraa R., Alharbi A., Ossra W. (2023). The Prevalence of Positional Obstructive Sleep Apnoea in a Sample of the Saudi Population. J. Epidemiol. Glob. Health.

[57] Zuberi N.A., Rekab K., Nguyen H.V. (2004). Sleep apnea avoidance pillow effects on obstructive sleep apnea syndrome and snoring. Sleep Breath..

[58] Bidarian-Moniri A., Nilsson M., Attia J., Ejnell H. (2015). Mattress and pillow for prone positioning for treatment of obstructive sleep apnoea. Acta Otolaryngol..

[59] Levendowski D.J., Seagraves S., Popovic D., Westbrook P.R. (2014). Assessment of a neck-based treatment and monitoring device for positional obstructive sleep apnea. J. Clin. Sleep Med..

[60] Van Maanen J.P., Meester K.A.W., Dun L.N., Koutsourelakis I., Witte B.I., Laman D.M., Hilgevoord A.A.J., De Vries N. (2013). The sleep position trainer: A new treatment for positional obstructive sleep apnoea. Sleep Breath..

[61] Yingjuan M., Siang W.H., Leong Alvin T.K., Poh H.P. (2019). Positional Therapy for Positional Obstructive Sleep Apnea. Sleep Med. Clin..

[62] Hidalgo Armas L., Ingles S., Vaca R., Cordero-Guevara J., Duran Carro J., Ullate J., Barbé F., Durán-Cantolla J. (2021). New forehead device in positional obstructive sleep apnoea: A randomised clinical trial. Thorax.

[63] Van Maanen J.P., Richard W., Van Kesteren E.R., Ravesloot M.J.L., Laman D.M., Hilgevoord A.A.J., De Vries N. (2012). Evaluation of a new simple treatment for positional sleep apnoea patients. J. Sleep Res..

[64] Laub R.R., Tønnesen P., Jennum P.J. (2017). A Sleep Position Trainer for positional sleep apnea: A randomized, controlled trial. J. Sleep Res..

[65] Eijsvogel M.M., Ubbink R., Dekker J., Oppersma E., De Jongh F.H., Van Der Palen J., Brusse-Keizer M.G. (2015). Sleep position trainer versus tennis ball technique in positional obstructive sleep apnea syndrome. J. Clin. Sleep Med..

[66] AlQarni A.S., Turnbull C.D., Morrell M.J., Kelly J.L. (2023). Efficacy of vibrotactile positional therapy devices on patients with positional obstructive sleep apnoea: A systematic review and meta-analysis. Thorax.

[67] Gao Y., Zhu S., Li W., Lai Y. (2025). Comparative efficacy of sleep positional therapy, oral appliance therapy, and CPAP in obstructive sleep apnea: A meta-analysis of mean changes in key outcomes. Front. Med..

[68] Patel S., Kon S.S.C., Nolan C.M., Barker R.E., Simonds A.K., Morrell M.J., Man W.D.C. (2018). The epworth sleepiness scale: Minimum clinically important difference in obstructive sleep apnea. Am. J. Respir. Crit. Care Med..

[69] Weaver T.E., Menno D.M., Bron M., Crosby R.D., Morris S., Mathias S.D. (2021). Determination of thresholds for minimally important difference and clinically important response on the functional outcomes of sleep questionnaire short version in adults with narcolepsy or obstructive sleep apnea. Sleep Breath..

[70] Permut I., Diaz-Abad M., Chatila W., Crocetti J., Gaughan J.P., D’Alonzo G.E., Krachman S.L. (2010). Comparison of Positional Therapy to CPAP in Patients with Positional Obstructive Sleep Apnea. J. Clin. Sleep Med..

[71] POSA Trial. NCT04153240.

[72] Rahimi M.M., Vakulin A., McEvoy R.D., Barnes M., Quinn S.J., Mercer J.D., O’Grady A., Antic N.A., Catcheside P.G. (2024). Comparative Effectiveness of Supine Avoidance versus Continuous Positive Airway Pressure for Treating Supine-isolated Sleep Apnea: A Clinical Trial. Ann. Am. Thorac. Soc..

[73] Benoist L., de Ruiter M., de Lange J., de Vries N. (2017). A randomized, controlled trial of positional therapy versus oral appliance therapy for position-dependent sleep apnea. Sleep Med..

[74] Mohamed A.M.A., Mohammed O.M., Liu S., Al-balaa M., Al-warafi L.A., Peng S.J., Qiao Y.Q. (2024). Oral appliance therapy vs. positional therapy for managing positional obstructive sleep apnea; a systematic review and meta-analysis of randomized control trials. BMC Oral Health.

[75] Suzuki M., Funayama Y., Homma M., Shibasaki K., Furukawa T., Yosizawa T. (2021). Effect of position therapy and oral devices on sleep parameters in patients with obstructive sleep apnea. Eur. Arch. Oto-Rhino-Laryngol..

[76] Marciuc D., Morarasu S., Morarasu B.C., Marciuc E.A., Dobrovat B.I., Pintiliciuc-Serban V., Popescu R.M., Bida F.C., Munteanu V., Haba D. (2023). Dental Appliances for the Treatment of Obstructive Sleep Apnea in Children: A Systematic Review and Meta-Analysis. Medicina.

[77] Marklund M., Stenlund H., Franklin K.A. (2004). Mandibular advancement devices in 630 men and women with obstructive sleep apnea and snoring: Tolerability and predictors of treatment success. Chest.

[78] Makihara E., Watanabe T., Ogusu H., Masumi S.I. (2022). The comparison of two different mandibular positions for oral appliance therapy in patients with obstructive sleep apnea. Clin. Exp. Dent. Res..

[79] Jokic R., Klimaszewski A., Crossley M., Sridhar G., Fitzpatrick M.F. (1999). Positional Treatment vs Continuous Positive Airway Pressure in Patients With Positional Obstructive Sleep Apnea Syndrome. Chest.

[80] Sagiraju R., Ramzy J., Cohen D., Kaur P., Kim S., Espinoza E., Zheng M. (2025). 0750 Efficacy of Hypoglossal Nerve Stimulation in Positional and Non-Positional Obstructive Sleep Apnea. Sleep.

[81] White M., Stuewe E., Zacharias R., Grover A., Wein R. (2021). 461 Hypoglossal Nerve Stimulation: Effectiveness of Therapy for Treatment of Positional Obstructive Sleep Apnea. Sleep.

[82] Dieltjens M., Vroegop A.V., Verbruggen A.E., Wouters K., Willemen M., De Backer W.A., Verbraecken J.A., Van de Heyning P.H., Braem M.J., de Vries N. (2014). A promising concept of combination therapy for positional obstructive sleep apnea. Sleep Breath..

[83] Ravesloot M.J.L., White D., Heinzer R., Oksenberg A., Pépin J.L. (2017). Efficacy of the New Generation of Devices for Positional Therapy for Patients With Positional Obstructive Sleep Apnea: A Systematic Review of the Literature and Meta-Analysis. J. Clin. Sleep Med..

[84] Van Maanen J.P., De Vries N. (2014). Long-Term Effectiveness and Compliance of Positional Therapy with the Sleep Position Trainer in the Treatment of Positional Obstructive Sleep Apnea Syndrome. Sleep.

[85] de Corso E., Mastrapasqua R.F., Fiorita A., Settimi S., Mele D.A., Picciotti P.M., Loperfido A., Marrone S., Rizzotto G., Paludetti G. (2020). Efficacy and long-term follow-up of positional therapy by vibrotactile neck-based device in the management of positional OSA. J. Clin. Sleep Med..

[86] Vanderveken O.M., Dieltjens M., Wouters K., De Backer W.A., Van De Heyning P.H., Braem M.J. (2013). Objective measurement of compliance during oral appliance therapy for sleep-disordered breathing. Thorax.

[87] Grote L., Hedner J., Grunstein R., Kraiczi H. (2000). Therapy with nCPAP: Incomplete elimination of Sleep Related Breathing Disorder. Eur. Respir. J..

[88] Poletti V., Pagnini F., Banfi P., Volpato E. (2023). Illness Perceptions, Cognitions, and Beliefs on COPD Patients’ Adherence to Treatment—A Systematic Review. Patient Prefer. Adherence.

[89] Poletti V., Bresciani G., Banfi P., Volpato E. (2024). Exploring perceptions and expectations of COPD patients: A grounded theory approach for personalized therapeutic interventions. Chronic Respir. Dis..

